# Impact of temperature, feeding preference and vaccination on Schmallenberg virus transmission in Scotland

**DOI:** 10.1038/srep05746

**Published:** 2014-07-18

**Authors:** Paul R. Bessell, Harriet K. Auty, Kate R. Searle, Ian G. Handel, Bethan V. Purse, B. Mark de C. Bronsvoort

**Affiliations:** 1 The Roslin Institute, The University of Edinburgh, Easter Bush, EH25 9RG; 2 Epidemiology Research Unit, SRUC, Drummond Hill, Stratherrick Road, Inverness, IV2 4JZ; 3 Centre for Ecology and Hydrology, Bush Estate, Edinburgh, EH26 0QB; 4 Centre for Ecology and Hydrology, Maclean Building, Benson Lane, Crowmarsh Gifford, Wallingford, OX10 8BB

## Abstract

First identified in 2011, Schmallenberg virus (SBV) is principally transmitted by *Culicoides* midges and affects ruminants. Clinical presentation is typified by foetal abnormalities, but despite very high infection rates, relatively few animals present with clinical signs. In this paper we further develop a previously published stochastic mathematical model of SBV spread to investigate the optimal deployment of a vaccine for SBV in Scotland, a country that has experienced only sporadic and isolated cases of SBV. We consider the use of the vaccine under different temperatures and explore the effects of a vector preference for feeding on cattle. We demonstrate that vaccine impact is optimised by targeting it at the high risk areas in the south of Scotland, or vaccinating only cattle. At higher than average temperatures, and hence increased transmission potential, the relative impact of vaccination is considerably enhanced. Vaccine impact is also enhanced if vectors feed preferentially on cattle. These findings are of considerable importance when planning control strategies for SBV and also have important implications for management of other arboviruses such as Bluetongue virus. Environmental determinants and feeding preferences should be researched further to inform development of effective control strategies.

Schmallenberg virus (SBV) is a novel Orthobunyavirus, a member of the Simbu serogroup and is closely related to Akabane and Shamonda viruses^1^.

SBV emerged in late 2011 with clinical signs that are characterised by pyrexia, reduced milk production, abortions and congenital malformations among offspring whose mothers are infected during a particular period of pregnancy^1^,^2^. During 2011 and 2012 SBV spread widely in the cattle, sheep and goat populations of Western Europe, with 8,730 herds and flocks reported infected by May 2013^3^. However, cases are typically only identified when or if animals present with birth malformations and this lack of overt clinical presentation is a principal reason that there is a large degree of under-ascertainment for SBV^4^,^5^,^6^. Malformations in births are thought to be most likely if ewes are infected between days 28 and 56 of pregnancy, or cows infected between days 60 and 172 of pregnancy, but these at risk periods still require more specific characterisation for SBV^7^.

The main route of transmission is via arthropod vectors (principally midges of *Culicoides* spp)^8^,^9^,^10^,^11^, Schmallenberg virus has also been identified in the semen of bulls but venereal transmission has not yet been demonstrated^12^,^13^,^14^. Vertical transmission within hosts has been observed but is not considered important in disease spread^15^. The reliance on midge vectors for disease transmission means that disease spread is limited seasonally by the duration of the adult vector season^16^. There is evidence that vector transmission can occur even when animals are housed indoors^17^. That there is evidence of spread during 2011, 2012 and 2013 suggests that the disease has mechanisms for overwintering through the period of low vector activity. The mechanisms for overwintering are yet to be determined but could be through vertical transmission, vector survival in indoor animal housing, or some other mechanism.

To date, there have been few clinical cases reported in Scotland, and this has been supported by active case seeking through bulk milk sampling and postal surveys^18^,^19^. Additionally, a survey found very low seroprevalences for SBV in northern England, in contrast to considerably higher seroprevalences in southern England^20^. The recent decline in incidence across Europe and the low seroprevalences in northern England suggest that SBV may have burned out^5^, but the possibility of reintroduction into these serologically naive populations remains.

A recent study modelled the likely impacts of SBV following introduction into Scotland^21^, a country that saw only sporadic cases of SBV during 2012 and 2013^22^,^23^,^24^. The study found that the ability of the virus to spread within Scotland is highly sensitive to the temperature as it determines the incubation within the vector. Of particular importance were the ambient temperature, and the Extrinsic Incubation Period (EIP), which is the length of time between the vector becoming infected and infectious. Since that publication, which used the EIP of BTV in the absence of SBV specific information, the EIP for SBV has been characterised and indicates that SBV is capable of incubating both at lower temperatures and at faster rate that was observed for BTV^25^,^26^. Furthermore, it has previously been assumed, including in the study by Bessell *et al*.^21^ that *Culicoides* feed with equal likelihood on a cow compared to a sheep. A number of studies have explored the species feeding preferences for *Culicoides* midges. Generally *Culicoides* midges have a wide host range and tend to blood feed opportunistically^27^,^28^,^29^,^30^ but for some European species, it has been suggested that they preferentially feed on cattle rather than sheep. For example, Ayllón *et al.* (2014) demonstrated that a midge is around 5 times more likely to feed on a cow compared to a sheep^31^. This finding could have implications for the likely pattern of transmission for a highly infectious disease such as SBV in mixed livestock contexts.

Vaccines against SBV are now available in the UK. Although full efficacy data are not yet available, the efficacy is reported to be very high^32^. SBV is not a notifiable disease in the UK and it is likely that farmers would be given the choice of whether to vaccinate their animals. Therefore guidance as to how to use the vaccine most effectively would be beneficial.

Given the further data that now exist on SBV transmission, and the availability of vaccines, the aims of this paper are:

1. To evaluate the effect of temperature on the potential for SBV spread in Scotland;
2. To evaluate the potential for vector feeding preferences to alter the pattern of SBV transmission;
3. To evaluate the effectiveness of different strategies for targeting vaccines for SBV;
4. To evaluate the potential for spread in subsequent years following the introduction of SBV.

## Results

### Temperature

**Figure 1:**
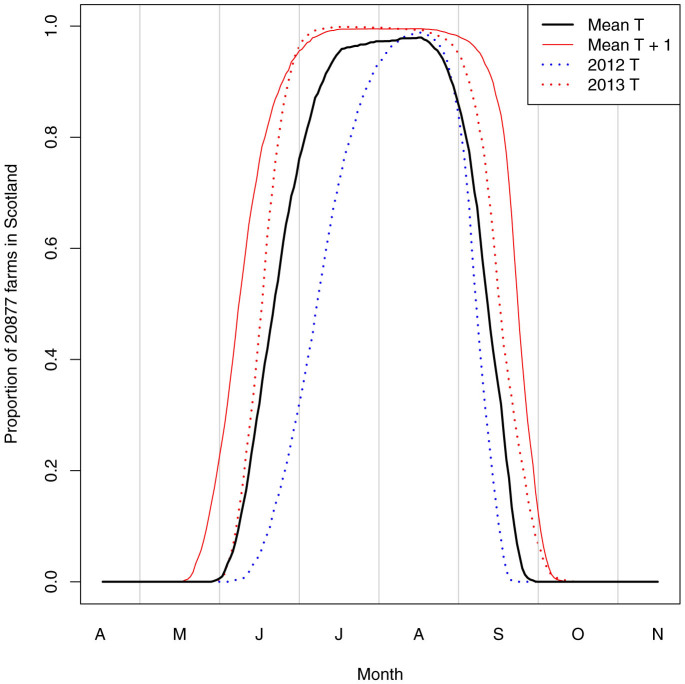
The proportion of farms for which the daily average temperature is greater than the baseline for the EIP of 12.35°C during the summer months. This is presented for the mean temperature, mean temperature +1°C, 2012 mean temperature and 2013 mean temperature.

The peak number of infections occurs when the disease is introduced on day 45 of the vector season, corresponding to the 15^th^ June, except when temperatures are 2°C above the daily mean for 1990–2006 (Table 1). At lower temperatures closer to the daily mean for 1990–2006 and temperatures seen during 2012, many introductions do not result in spread if the introduction is before the 15^th^ June, this is due to the temperature being too low for incubation in the vector (Table 1; Figure 1). Whilst temperatures are still sufficiently high for incubation during July and August, there is less time remaining for disease transmission, hence the marked decrease in the number of infections despite the epidemic reliably taking off during these times.

**Figure 2:**
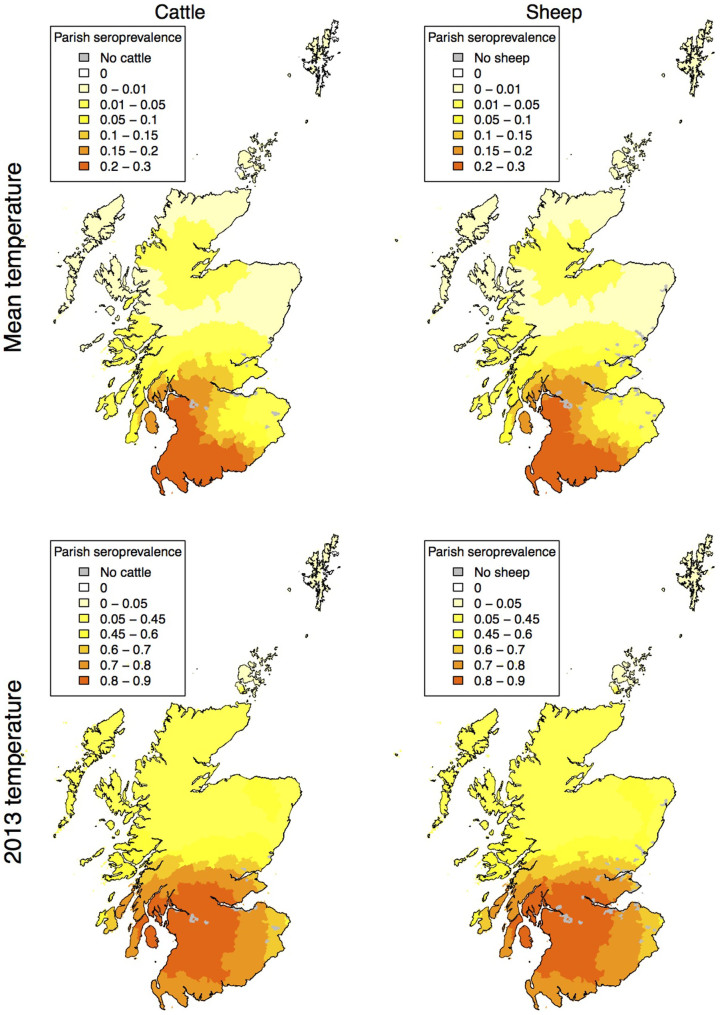
The parish level seroprevalence as a proportion of animals infected (averaged over 1000 model iterations) among cattle and sheep following introduction on day 45 given the mean temperature (top) and the temperatures seen during 2013 (bottom). Maps created using R^33^,^34^.

The pattern of infections shows a focus in the south west for both cattle and sheep, this applies in years with daily mean temperatures and when the temperatures match those from 2013 (Figure 2).

**Figure 3:**
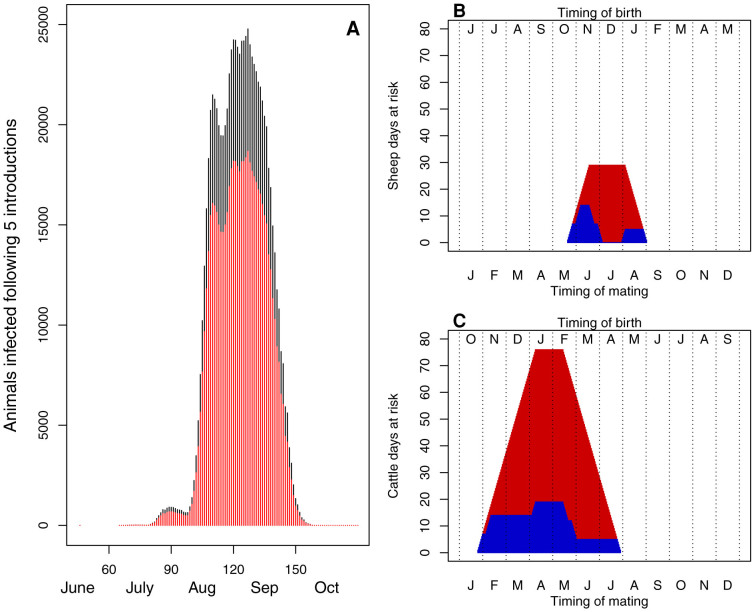
Given the epidemic in Figure 3A which is based upon the epidemic fitted to the mean temperature in Figure 2, graphs B and C show the number of days of pregnancy that a cow or ewe will be at risk of infection that could lead to birth abnormalities, depending on the timing of mating. The red bars represent elevated risk (greater than 0.01% of animals infected daily) and the blue blocks lower (but still some) risk (greater than 0.001% and less than 0.001% of animals infected daily).

In an epidemic with the daily mean temperatures for 1990–2006 and an introduction on day 45, it is only sheep flocks that lamb prior to February that would be at considerable risk of presenting with birth malformations. The window in which cattle would be at risk is larger, this is due to the longer window of pregnancy in which cattle are at risk (Figure 3).

### Feeding preferences

**Figure 4:**
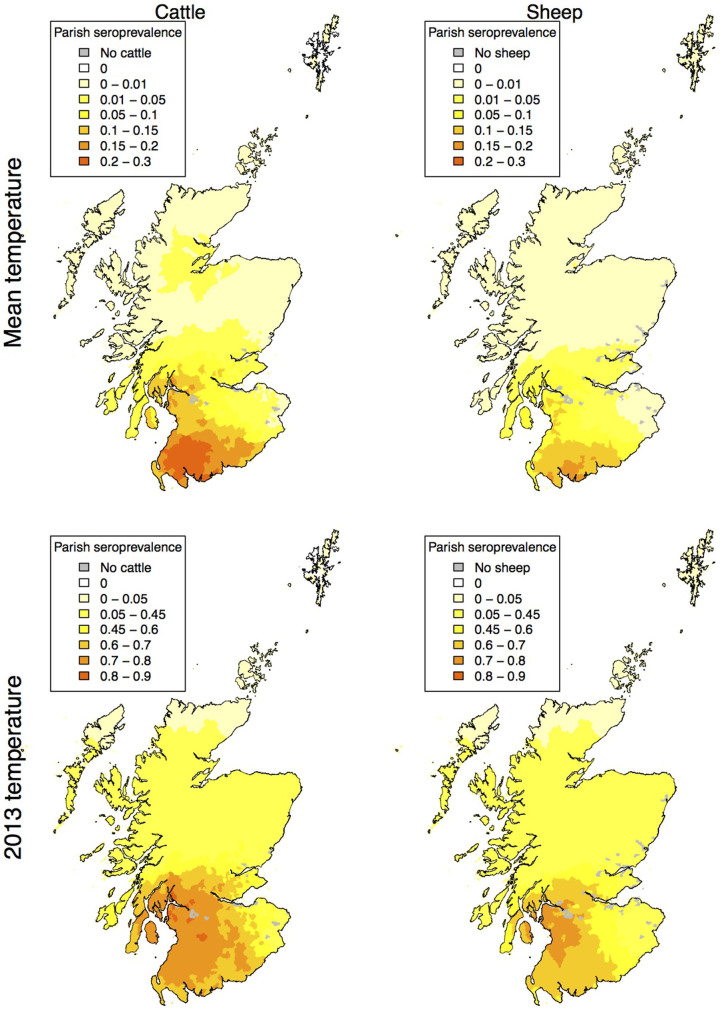
Map of SBV seroprevalence as a proportion of animals infected (averaged over 1000 model iterations) when there is a vector preference for feeding upon cattle rather than sheep. Maps are shown for mean temperatures (top) and temperatures experienced during 2013 (bottom). Maps created using R^33^,^34^.

If *Culicoides* have a preference for feeding on cattle rather than sheep, the pattern of infections changes (Figure 4). The high incidence areas of both cattle and sheep shrink but the higher incidence area for sheep shrinks more than that of cattle and the impact of the decline in infections is greater in cooler years (Table 2).

**Figure 5:**
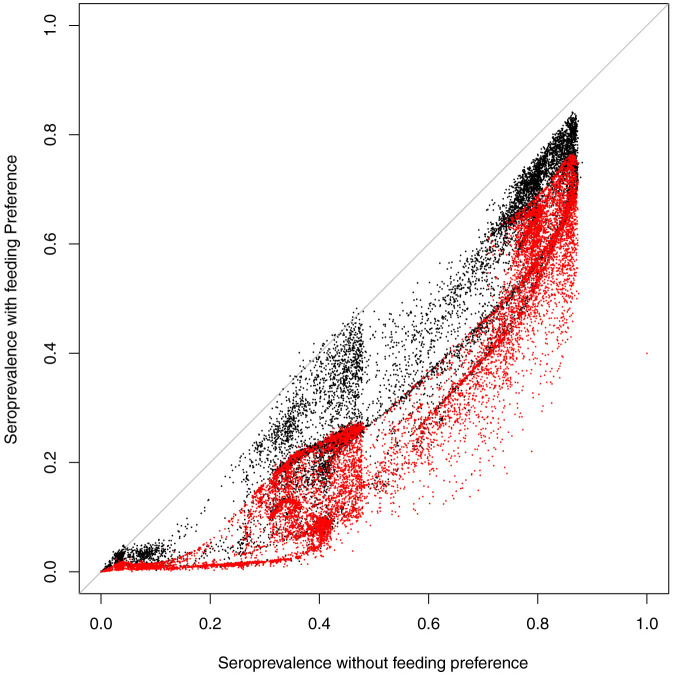
Farm level seroprevalence for cattle (black points) and sheep (red points) with and without a vector feeding preference. This is fitted with mean temperatures from 2013.

In Scotland 8,297 (39.7%) of susceptible farms have only sheep, 6,058 (29.0%) have only cattle and 6,522 (31.2%) have both species, but nearly all farms show a decline in seroprevalence when midges show a feeding preference towards cattle rather than sheep. This is associated with the general decline in transmission brought about by reduced biting rates on sheep (Figure 5). Furthermore, Figure 5 shows distinct clusters at different seroprevalences that correspond to the geographic clusters (Figure 2 and 4) corresponding to the south west (centred around 0.8 on the x- and 0.7 on the y- axis) and the north of Scotland (centred around 0.4 on the x- and 0.15 on the y-axis).

### Vaccination

Measured in terms of the number of additional animals that are protected by each vaccinated animal, vaccination has the greatest impact on disease transmission when it is targeted at animals in the south or specifically at cattle, rather than implemented on an *ad hoc* basis. This is particularly the case in higher temperatures. During a year of average temperatures, one vaccinated animal under the “south” or “cattle” implementations results in 0.243 or 0.301 fewer infections per vaccinated animal respectively. When temperatures are 1°C higher these increase to 0.894 and 0.621 respectively, thus both the vaccinated animal plus a proportion of unvaccinated stock are protected from potential birth malformations. The effects of vaccination also last into subsequent years and are amplified when the vector has a preference for feeding upon cattle rather than sheep (Tables 3 and 4).

**Figure 6:**
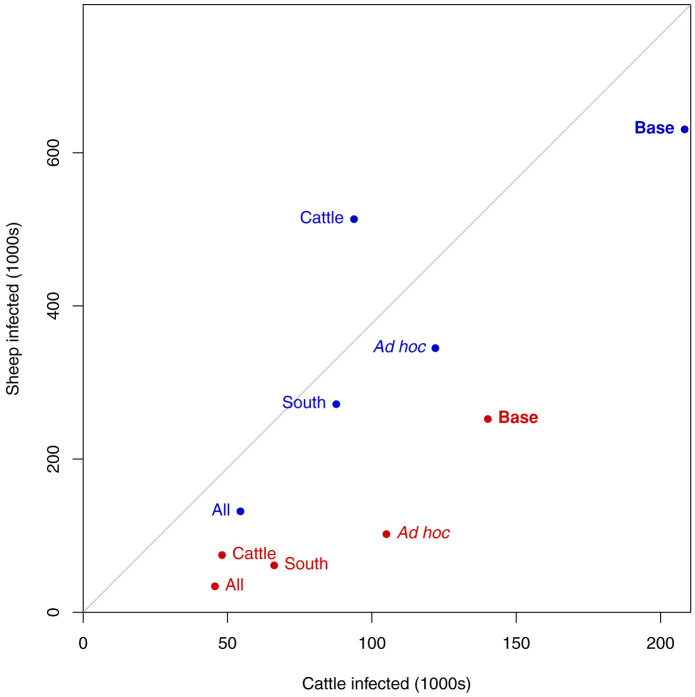
The numbers of infected animals resulting from disease introduction under different vaccination strategies. The red points are vaccination with a vector feeding preference for cattle and blue points the vaccination strategy without feeding preference. “Base” refers to no vaccination. The grey centre line is the equality line – if sheep and cattle were becoming infected at equal rates relative to their population.

With a cattle feeding preference, vaccinating all animals has the greatest impact upon reducing infections in both cattle and sheep, but the sheep that are vaccinated in this strategy have a relatively small impact. If just cattle are vaccinated, the impact is similar, a similar number of cattle infected resulted in a slight increase in the number of sheep infected compared to the “All” strategy (Figure 6).

## Discussion

These analyses have demonstrated that there is considerable potential for SBV to spread in Scotland, and that this potential is orders of magnitude greater than was previously modelled using the longer EIP in the analysis of Bessell *et al.*^21^. Like the results of Bessell *et al.*^21^ these results demonstrate that the potential for spread is highly sensitive to both the temperature and the timing of introduction (Table 1). During a year of average temperatures the disease must be introduced during the month of June for there to be substantial spread. This has implications for the options for control of the disease, so the timing of introduction could be coupled with data on the current temperatures to give an early warning as to the threat that the introduction presents. This would provide the opportunity to develop appropriate control options based upon these data. In this case, the model could be further adapted to incorporate temperature and precipitation in the kernel shape.

Furthermore, these results have demonstrated that during 2012, a cooler year relative to the mean, there was little potential for the spread of SBV in Scotland. This was the year when there was the greatest amount of SBV spread in Europe and England and these results may explain the small number of cases that occurred in Scotland^5^,^20^. It may be the case that there were many (undetected) introductions of SBV into Scotland during 2012, but that the temperatures were simply too low for spread to be possible or the introductions were too late in the season. Had the disease been spreading and been introduced during 2013 then there may have been considerable spread of SBV in Scotland. That there was not more SBV spread during 2013 indicates that there was no introduction during a period that would have resulted in spread (Table 1). Reports from Europe and England indicate that there was relatively little SBV circulation during June and July^5^ when an introduction into Scotland would have resulted in spread. Correspondingly, the window in which sheep are at risk of birth malformations is relatively narrow and in Scotland SBV will only be a threat to those flocks that lamb relatively early (January lambing flocks would be considered early lambing and are relatively few in Scotland). The period over which cattle are likely to be exposed to infection during the at-risk period of pregnancy is considerably greater. However a study has indicated that the risks of cattle developing birth malformations is considerably lower than for sheep^6^.

Vaccination can be used by individual livestock owners with the aim of protecting their animals from infection that may cause losses (represented by the *ad hoc* vaccination strategy in this study). Alternatively, vaccination may be used more tactically to break the transmission chain and reduce overall disease spread. In this study, tactical vaccination was represented by the strategies of vaccination in the south and vaccinating cattle. Although *ad hoc* vaccination is beneficial to individual livestock keepers, our study clearly shows the overall benefit of using tactical approaches, particularly vaccination of cattle only. The value of the cattle vaccination strategy is further enhanced if there is a cattle feeding preference.

The importance of vaccination of cattle increases when a preference for *Culicoides* feeding on cattle is incorporated in the model. This narrows the chain of transmission as the vector is feeding preferentially on animals that are likely to have been protected. The cattle feeding preference reduces the number of sheep infected, but also in many cases the number of cattle infected (Figure 3). This is also due to breaking the transmission chain by infected vectors repeatedly feeding on animals that have previously been infected. Due to the greater force of infection at higher temperatures the effect of feeding preference diminished at higher temperatures (Tables 3 and 4). In common with other midge borne pathogens, vector feeding preferences have a significant effect on SBV transmission. This emphasizes the importance of field studies in providing data to refine these model parameters. Previous modelling studies of BTV in Great Britain included a feeding preference but did not explicitly consider its impact^35^, and just one study from elsewhere considers feeding preferences and BTV^36^. For African Horse Sickness virus infections feeding preferences have been demonstrated to have a large impact on disease transmission^37^. Whilst some field studies have found a midge feeding preference for cattle^27^,^28^,^29^,^30^, evidence across host feeding studies is variable, usually indicating that livestock-associated species are opportunistic and will feed on any available large mammals in their vicinity. Several livestock associated species in Europe feed on wild deer and domestic ruminants (in the same site) particularly in woodland and extensive pasture contexts^29^,^38^,^39^. More host preference studies would be valuable to explicitly quantify available hosts^30^ and to examine how preferences vary between species and landscape contexts.

As temperature increases, the potential for spread in subsequent years decreases. This is because the animals become infected and develop immunity during the first year and thus fewer susceptible animals remain. The lack of ongoing cases in Europe, where average temperatures are higher, may be explained by this depletion of susceptible animals.

These results are important for SBV management and control, but also have important implications should there be another epidemic of BTV in GB. As illustrated here for SBV, incorporation of more accurate knowledge on vector feeding preferences into models for BTV may provide an opportunity to break the transmission chain by targeting vaccination at animals at greatest risk of infection. In the face of an epidemic, such information may be very helpful in prioritising resources to limit spread.

## Methods

This analysis is carried out through extension of the spatially explicit stochastic model described by Bessell *et al*.^21^. This model incorporates the spatial livestock distribution from the Scottish agricultural census and historical temperature records. These animals are fed on by vectors at a vector-host ratio that is defined by the time of year and land cover in the neighbourhood of the farm. Infection will pass from animal to vector with a probability of 0.19^11^. If infected, the vector will survive to lay a potentially infectious bite on another animal by a probability defined by the vector mortality rate and EIP. The EIP, interval between blood meals and vector mortality rate are temperature dependent parameters, for the EIP there is a minimum temperature below which the process will not operate. The infected vector will feed on animals on the same or other farms as defined by a Gaussian spatial kernel with a mean transmission distance of 14 km^35^ that describes both the movement of the vector and movement of infected livestock. The model assumes a season of vector activity that runs between 1^st^ May and 31^st^ October with two peaks in vector abundance.

### Model implementation

The model is initiated by introduction of infection on to five randomly selected farms and running 1000 independent iterations of the model. Separate model implementations seed infections on 6 different start days starting on 16^th^ May at 15 day intervals. The paper of Bessell *et al.*^21^ explored the potential sensitivity of disease spread to temperature variations and of adjusting the threshold minimum temperature required for the EIP to be completed. The EIP parameter is defined by: 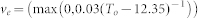


where T_0 _is the observed temperature and 12.35°C is the minimum temperature at which incubation can take place^25^.

In this model, T_O_ is the daily average temperature on each farm extrapolated from the Met Office UKCIP archive of temperature data that were interpolated and gridded to 5 km^2^ cells and averaged over the years between 1990 and 2006^40^. To explore the effect of temperature variation on disease transmission and its impact of vaccination (i) we add 0.5°C, 1°C and 2°C to T_O_; and (ii) we adjust temperatures by monthly anomalies (the deviation from the mean temperature) in 2012 and 2013 (Figure 1). In general, temperatures in 2012 were 0.8°C lower than average, and in 2013 were 0.92°C higher than average.

We go on to consider the potential for spread given reintroduction of the virus during the following year. To incorporate stock replacement and the introduction of new, serologically naïve stock, a proportion of the livestock population in the model is replaced annually. Amongst sheep 49% of stock is replaced. This comprises replacement of breeding stock over one year old (22.2%) and slaughter of animals under one year for consumption (77.8%) (data from the Scottish agricultural census^41^). Among cattle 27.5% of stock is replaced; 55% of the stock is under 2 years old and 31.9% of these animals are slaughtered annually (data from the Cattle Tracing System cattle movement database). 18.4% of the animals over two years old are slaughtered and replaced from the younger stock annually.

### Feeding preferences

In all analyses we consider two scenarios for feeding preference:

1. The vector is equally likely to feed on a cow as a sheep.
2. The odds that a midge will feed on a sheep with odds of 0.205 relative to a cow, based upon Ayllón *et al.*^31^.

### Vaccination

Assuming 100% efficacy, we consider different deployments of the vaccine aimed at minimising reproductive losses and reducing virus transmission. We consider vaccination prior to the start of the first vector season (first year of disease spread) and we only consider vaccinating animals that are likely to be used for breeding replacement animals. This comprises 45% of Scottish cattle and 51% of Scottish sheep^41^. The model considers four strategies for deploying the vaccine:

1. All cattle and sheep that are being reared for breeding replacement stock are vaccinated (referred to in the results as “all”);
2. 50% of herds and flocks (selected randomly) vaccinate all breeding cattle and sheep, to represent a voluntary program with 50% uptake (“*ad hoc* vaccination”);
3. All breeding cattle are vaccinated (“cattle vaccination”);
4. Breeding cattle and sheep on herds and flocks located in the south of Scotland, in the counties to the south of the Forth and Clyde are vaccinated (“south vaccination”);

We analyse the protective impact of vaccination by comparing the results of model runs with vaccination against baseline scenarios in which there is no vaccination undertaken. We evaluate:

1. The number of animals vaccinated and percentage of animals vaccinated.
2. The percentage reduction in the number of infections. Calculated as: 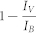 where I_B_ is the number infected without vaccination and I_V_ is the number infected with vaccination.
3. The number of infections spared per vaccinated animal. Calculated as: 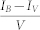 where I_B_ is the number infected without vaccination, I_V_ is the number infected with vaccination and V the number vaccinated.

### Model assumptions

There are a number of assumptions that underlie this paper, some of which have previously been stated in Bessell *et al.*^21^:

1. Farm composition is homogeneous and ages of animals on each farm are assumed to be the same across the country. Specifically, that different production types, such as beef and dairy farms, or hill and lowland sheep farms can be treated similarly. Potentially, if the spatial distribution of an epidemic were being analysed the homogeneity of farms may create more specific foci of spread.
2. *Culicoides* will blood feed on young animals with equal probability as an adult animal. Based upon experiments exploring the attraction of *Culicoides* to carbon dioxide^42^,^43^ it remains a possibility that *Culicoides* are more attracted to adult rather than juvenile animals.
3. Movements of exposed or infectious animals are not explicitly considered, although they are incorporated in the transmission kernel^35^.
4. The range of dispersal of the vector can be modelled using a kernel. Vector dispersal is influenced by many factors including weather^44^,^45^. However, localised wind patterns are difficult to model in the long term and it has been demonstrated elsewhere that during periods of intense midge activity BTV transmission behaves in a similar manner to direct transmission^46^ suggesting a kernel is a suitable approximation.
5. That the width of the spatial kernel will not vary with temperature. Given a daily *Culicoides* dispersal distance, the transmission kernel should vary as the incubation period varies.
6. The animal is equally infectious on each day of its infectious period.
7. Once infected an animal will recover with full immunity and will not be susceptible to further infection.
8. The attractiveness of a farm for vector feeding is based on the number of livestock on the farm and is determined by distance and the number of livestock.
9. SBV vaccines have an efficacy of 100%. Large studies of efficacy are not yet available.
10. We do not incorporate vectors feeding on species other than cattle and sheep. Other hosts include horses and wild ruminants, but the distribution of these species and the vector ecology in terms of feeding is relatively poorly understood.
11. Scotland can be regarded in isolation. During an epidemic involving southern Scotland there is likely to be some transmission with farms in northern England. However, as this is likely to be a two-way exchange we consider that this would have minimal effect on the epidemic.

## Author Contributions

P.B., M.B. and I.H. conceived and designed the study, P.B. performed the analysis and drafted the manuscript, K.S. and B.P. contributed to aspects of arbovirus epidemiology and H.A. contributed to veterinary aspects of SBV. All authors have read, made changes to and approved the manuscript.

## Figures and Tables

**Table 1:** (Top half) The number of animals infected under these temperature scenarios and (bottom half) the percentage of introductions that result in more than 10 infected animals (5 introductions and 5 subsequent infections) during one completed vector season given introductions on different days

Temperature	Mean number of animals infected given date of introduction					
16^th^ May	31^st^ May	15^th^ June	30^th^ June	15^th^ July	30^th^ July	
**Mean temp**	5.02	384,668	839,439	485,762	5,829	805
**Mean temp +0.5°C**	58,490	2,404,203	3,274,581	1,906,485	95,896	5,393
**Mean temp +1°C**	1,234,702	5,335,513	5,891,231	4,741,308	557,263	31,123
**Mean temp +2°C**	7,034,805	8,011,970	7,938,294	7,449,821	4,974,377	1,473,589
**2012**	5	1,088	47,970	49,541	2,892	506
**2013**	5.02	2,164,895	5,210,915	3,827,855	139,002	4,422

Temperature	% R_0_ (first generation) > 1					
16^th^ May	31^st^ May	15^th^ June	30^th^ June	15^th^ July	30^th^ July	
**Mean temp**	0.2	67.8	98.9	100	99.8	99.6
**Mean temp +0.5°C**	14.3	100	100	100	100	100
**Mean temp +1°C**	51	99	100	100	100	100
**Mean temp +2°C**	99	100	100	100	100	100
**2012**	0	3.8	57.2	98.2	100	100
**2013**	0	88.2	100	100	100	100

**Table 2:** The number of animals infected with and without a feeding preference for cattle under the mean temperature and the mean temperature increased by 1°C

		Cattle infected	Sheep infected
Mean	Baseline	207,945	631,493
temperature	Feeding preference	137,786	244,069
	% reduction	33.7	61.4
Mean	Baseline	1,272,249	4,618,982
temperature	Feeding preference	1,049,441	3,207,307
+1°C	% reduction	17.5	30.6

**Table 3:** The effects of vaccination under mean temperatures with and without a vector feeding preference for cattle

	Year 1			Years 1 and 2 combined	
Breeding stock vaccinated	No. vaccinated (%)	% Reduction	Vaccinates: Infections prevented	% Reduction	Vaccinates: Infections prevented
All	4,294,102 (49.9)	77.8	0.152	69.8	0.240
South	2,067,943 (24.0)	57.1	0.232	49.0	0.350
*Ad hoc*	2,146,349 (24.9)	43.4	0.173	36.9	0.254
Cattle	817,671 (9.50)	27.6	0.283	19.1	0.345
**Cattle feeding preference**					
All	4,294,102 (49.9)	79.7	0.073	73.0	0.117
South	2,067,943 (24.0)	67.5	0.128	60.7	0.202
*Ad hoc*	2,146,349 (24.9)	47.3	0.086	40.1	0.128
Cattle	817,671 (9.50)	68.7	0.330	61.3	0.515

**Table 4:** The effects of vaccination when temperatures are 1°C warmer with and without a vector feeding preference for cattle

	Year 1			Years 1 and 2 combined	
Breeding stock vaccinated	No. vaccinated (%)	% Reduction	Vaccinates: Infections prevented	% Reduction	Vaccinates: Infections prevented
All	4,294,102 (49.9)	60.1	0.814	45.9	0.933
South	2,067,943 (24.0)	33.1	0.930	24.4	1.03
*Ad hoc*	2,146,349 (24.9)	29.5	0.798	22.8	0.927
Cattle	817,671 (9.50)	10.8	0.771	9.07	0.970
**Cattle feeding preference**					
All	4,294,102 (49.9)	68.8	0.687	60.3	0.862
South	2,067,943 (24.0)	46.5	0.963	40.5	1.20
*Ad hoc*	2,146,349 (24.9)	38.4	0.766	35.0	1.00
Cattle	817,671 (9.50)	46.9	2.46	39.1	2.94
